# Supplementary material for: Data-driven brain network models differentiate variability across language tasks
Source: PLoS Comput Biol. 2018 Oct 17;14(10):e1006487. doi: 10.1371/journal.pcbi.1006487 (PMC6192563; doi:10.1371/journal.pcbi.1006487)
Supplement: S1 Table — The variables r and p denote the Pearson correlation coefficient and associated p-value, respectively. The 90% confidence interval for r is reported below each correlation. Here a * denotes that the observed correlation is significant under FDR correction for multiple comparisons across tasks (for p < 0.05) and a • denotes a significant correlation across the two scales of the brain parcellation studied in this paper. VG = verb generation, SC = sentence completion, and NR = number reading. (DOCX) [file pcbi.1006487.s003.docx]

| Model feature | VG | | SC | | NR | |
| --- | --- | --- | --- | --- | --- | --- |
|  | *r* | *p* | *r* | *p* | *r* | *p* |
| Transition value | 0.21  [-0.33, 0.74] | 0.56 | **0.76***•  [0.48, 0.91] | **0.01** | 0.42  [-0.27, 0.76] | 0.22 |
| Functional effect (global brain) | 0.12  [-0.43, 0.68] | 0.73 | 0.55  [-0.02, 0.87] | 0.10 | 0.34  [-0.33, 0.79] | 0.34 |
| Functional effect (task circuit) | 0.39  [-0.17, 0.86] | 0.26 | 0.42  [-0.22, 0.85] | 0.23 | **0.65**•  [0.29, 0.87] | **0.04** |
| Functional effect (outside the task circuit) | 0.10  [-0.45, 0.66] | 0.78 | 0.52  [-0.05, 0.87] | 0.11 | 0.31  [-0.36, 0.78] | 0.38 |
