# Supplementary material for: Data-driven brain network models differentiate variability across language tasks
Source: PLoS Comput Biol. 2018 Oct 17;14(10):e1006487. doi: 10.1371/journal.pcbi.1006487 (PMC6192563; doi:10.1371/journal.pcbi.1006487)
Supplement: S2 Table — The variables r and p denote the Pearson correlation coefficient and associated p-value, respectively. The 90% confidence interval for r is reported below each correlation. Here a * denotes that the observed correlation is significant under FDR correction for multiple comparisons across tasks (for p < 0.05) and a • denotes significant correlation across the two scales of the brain parcellation studied in this paper. VG = verb generation, SC = sentence completion, and NR = number reading. (DOCX) [file pcbi.1006487.s004.docx]

| Model feature | VG | | SC | | NR | |
| --- | --- | --- | --- | --- | --- | --- |
|  | *r* | *p* | *r* | *p* | *r* | *p* |
| Transition value | 0.45  [0.31, 0.71] | 0.19 | **0.61**•  [0.20, 0.86] | **0.05** | 0.57  [0.39, 0.91] | 0.09 |
| Functional effect (global brain) | 0.38  [0.09, 0.74] | 0.28 | 0.56  [0.01, 0.88] | 0.09 | 0.37  [-0.11, 0.77] | 0.30 |
| Functional effect (task circuit) | 0.62  [0.44, 0.89] | 0.06 | 0.26  [-0.31, 0.73] | 0.47 | **0.67**•  [0.28, 0.94] | **0.03** |
| Functional effect (outside the task circuit) | 0.35  [0.03, 0.72] | 0.32 | 0.55  [-0.002, 0.89] | 0.10 | 0.33  [-0.15, 0.75] | 0.35 |
