# Supplementary material for: Data-driven brain network models differentiate variability across language tasks
Source: PLoS Comput Biol. 2018 Oct 17;14(10):e1006487. doi: 10.1371/journal.pcbi.1006487 (PMC6192563; doi:10.1371/journal.pcbi.1006487)
Supplement: S3 Table — The variables r and p denote the Pearson correlation coefficient and associated p-value, respectively. The 90% confidence interval for r is reported below each correlation. Here a * denotes that the observed correlation is significant under FDR correction for multiple comparisons across tasks (for p < 0.05) and a • denotes a significant correlation across the two scales of the brain parcellation studied in this paper. VG = verb generation, SC = sentence completion, and NR = number reading. (DOCX) [file pcbi.1006487.s005.docx]

| Network feature | Task performance | VG | | SC | | NR | |
| --- | --- | --- | --- | --- | --- | --- | --- |
|  |  | *r* | *p* | *r* | *p* | *r* | *p* |
| Average degree | Before-TMS | -0.19  [-0.73, 0.45] | 0.61 | **-0.85***•  [-0.94, -0.62] | **0.002** | -0.23  [-0.67, 0.58] | 0.52 |
|  | Post-TMS | -0.26  [-0.64, 0.15] | 0.47 | **-0.70**•  [-0.88, -0.30] | **0.02** | -0.34  [-0.82, 0.21] | 0.34 |
| Inverse spectral radius | Before-TMS | 0.17  [-0.40, 0.72] | 0.64 | **0.78***•  [0.49, 0.92] | **0.008** | 0.45  [-0.22, 0.76] | 0.19 |
|  | Post-TMS | 0.44  [0.23, 0.69] | 0.20 | **0.64**•  [0.24, 0.88] | **0.04** | 0.58  [0.39, 0.90] | 0.07 |
| Synchronizability | Before-TMS | -0.01  [-0.50, 0.75] | 0.98 | 0.45  [-0.05, 0.80] | 0.19 | 0.46  [0.05, 0.88] | 0.13 |
|  | Post-TMS | 0.28  [-0.14, 0.63] | 0.43 | 0.39  [-0.15, 0.85] | 0.25 | 0.56  [0.32, 0.88] | 0.09 |
