# Supplementary material for: Data-driven brain network models differentiate variability across language tasks
Source: PLoS Comput Biol. 2018 Oct 17;14(10):e1006487. doi: 10.1371/journal.pcbi.1006487 (PMC6192563; doi:10.1371/journal.pcbi.1006487)
Supplement: S4 Table — A list of anatomical regions used to construct anatomical brain networks. Matrix indices of a given anatomical region are indicated for both of the resolution scales used (83 or 234 regions). (DOCX) [file pcbi.1006487.s006.docx]

| **Right hemisphere** | | **Brain region** | **Left hemisphere** | |
| --- | --- | --- | --- | --- |
| 234-region | 83-region |  | 234-region | 83-region |
| 1-4 | 1 | Lateral orbitofrontal | 116-119 | 42 |
| 5 | 2 | Pars orbitalis | 120 | 43 |
| 6 | 3 | Frontal pole | 121 | 44 |
| 7-9 | 4 | Medial orbitofrontal | 122,123 | 45 |
| 10,11 | 5 | Pars triangularis | 124 | 46 |
| 12,13 | 6 | Pars opercularis | 125,126 | 47 |
| 14-19 | 7 | Rostral middle frontal | 127-132 | 48 |
| 20-27 | 8 | Superior frontal | 133-141 | 49 |
| 28-30 | 9 | Caudal middle frontal | 142-144 | 50 |
| 31-36 | 10 | Precentral | 145-152 | 51 |
| 37-39 | 11 | Paracentral | 153,154 | 52 |
| 40 | 12 | Rostral anterior cingulate | 155 | 53 |
| 41 | 13 | Caudal anterior cingulate | 156 | 54 |
| 42,43 | 14 | Posterior cingulate | 157,158 | 55 |
| 44 | 15 | Isthmus cingulate | 159 | 56 |
| 45-49 | 16 | Postcentral | 160-166 | 57 |
| 50-53 | 17 | Supramarginal | 167-171 | 58 |
| 54-60 | 18 | Superior parietal | 172-178 | 59 |
| 61-66 | 19 | Inferior parietal | 179-183 | 60 |
| 67-71 | 20 | Precuneus | 184-188 | 61 |
| 72-73 | 21 | Cuneus | 189 | 62 |
| 74-75 | 22 | Pericalcarine | 190 | 63 |
| 76-80 | 23 | Lateral occipital | 191-195 | 64 |
| 81-83 | 24 | Lingual | 196-199 | 65 |
| 84-87 | 25 | Fusiform | 200-203 | 66 |
| 88 | 26 | Parahippocampal | 204 | 67 |
| 89 | 27 | Entorhinal | 205 | 68 |
| 90 | 28 | Temporal pole | 206 | 69 |
| 91-94 | 29 | Inferior temporal | 207-210 | 70 |
| 95-98 | 30 | Middle temporal | 211-214 | 71 |
| 99 | 31 | Bank of the Superior Temporal Sulcus | 215,216 | 72 |
| 100-104 | 32 | Superior temporal | 217-221 | 73 |
| 105 | 33 | Transverse temporal | 222 | 74 |
| 106-108 | 34 | Insula | 223-226 | 75 |
| 109 | 35 | Thalamus proper | 227 | 76 |
| 110 | 36 | Caudate | 228 | 77 |
| 111 | 37 | Putamen | 229 | 78 |
| 112 | 38 | Pallidum | 230 | 79 |
| 113 | 39 | Accumbens area | 231 | 80 |
| 114 | 40 | Hyppocampus | 232 | 81 |
| 115 | 41 | Amygdala | 233 | 82 |
|  |  | Brainstem | 234 | 83 |
